# Dissociation of Motor Task-Induced Cortical Excitability and Pain Perception Changes in Healthy Volunteers

**DOI:** 10.1371/journal.pone.0034273

**Published:** 2012-03-28

**Authors:** Magdalena S. Volz, Mariana Mendonca, Fernando S. Pinheiro, Huashun Cui, Marcus Santana, Felipe Fregni

**Affiliations:** 1 Laboratory of Neuromodulation, Department of Physical Medicine & Rehabilitation, Spaulding Rehabilitation Hospital and Massachusetts General Hospital, Harvard Medical School, Boston, Massachusetts, United States of America; 2 Charité Center for Neurology, Neurosurgery and Psychiatry, Campus Charité Mitte, Charité - Universitätsmedizin Berlin, Berlin, Germany; University of Maryland, College Park, United States of America

## Abstract

**Background:**

There is evidence that interventions aiming at modulation of the motor cortex activity lead to pain reduction. In order to understand further the role of the motor cortex on pain modulation, we aimed to compare the behavioral (pressure pain threshold) and neurophysiological effects (transcranial magnetic stimulation (TMS) induced cortical excitability) across three different motor tasks.

**Methodology/Principal Findings:**

Fifteen healthy male subjects were enrolled in this randomized, controlled, blinded, cross-over designed study. Three different tasks were tested including motor learning with and without visual feedback, and simple hand movements. Cortical excitability was assessed using single and paired-pulse TMS measures such as resting motor threshold (RMT), motor-evoked potential (MEP), intracortical facilitation (ICF), short intracortical inhibition (SICI), and cortical silent period (CSP). All tasks showed significant reduction in pain perception represented by an increase in pressure pain threshold compared to the control condition (untrained hand). ANOVA indicated a difference among the three tasks regarding motor cortex excitability change. There was a significant increase in motor cortex excitability (as indexed by MEP increase and CSP shortening) for the simple hand movements.

**Conclusions/Significance:**

Although different motor tasks involving motor learning with and without visual feedback and simple hand movements appear to change pain perception similarly, it is likely that the neural mechanisms might not be the same as evidenced by differential effects in motor cortex excitability induced by these tasks. In addition, TMS-indexed motor excitability measures are not likely good markers to index the effects of motor-based tasks on pain perception in healthy subjects as other neural networks besides primary motor cortex might be involved with pain modulation during motor training.

## Introduction

Pain is a multidimensional, complex, subjective experience. According to the International Association for the Study of Pain (IASP), pain is an unpleasant sensory and emotional experience associated with actual or potential tissue damage [1]. Thus, treatment remains a major challenge for health professionals as its acute and chronic pathogenesis are not completely understood.

Recent evidence has shown that the primary motor cortex (M1) might be a useful therapeutic target for behavioral and non-pharmacological interventions such as invasive and non-invasive brain stimulation [2], [3], [4], [5], [6], [7]. The initial evidence comes from a previous study showing that deafferentation of the spinothalamic pathway results in hyperactivity of thalamic neurons, which can be inhibited solely by electrical stimulation of the M1 [8]. The idea is that stimulation of M1 can change thalamic excitability through thalamo-cortical pathways [8], [9]. There are many factors that may explain these effects. For instance, M1 activation may lead to activation of the GABAergic inhibitory system and the reduction of activity in the thalamus [3]. M1 might be therefore an “entry port” to modulate dysfunctional activity in pain-related neural networks [9], [10]. Moreover, activation of M1 and thalamus, which are known to be involved with the organization of movements, may be associated with improvement in motor control, an important factor for the interruption of the maintenance of pain [11], [12], [13]. In line with this knowledge, recent clinical findings have shown that activation of M1 with neuromodulatory techniques is efficient in reducing suffering in patients with chronic pain [6], [7], [14], [15], [16], [17]. M1 modulation via non-invasive brain stimulation may decrease thalamic hyperactivity and change neuronal plasticity, and these effects are also conveyed to other pain-related areas such as subthalamic areas, cingulate gyrus and spinal cord [18], [19], [20].

Other techniques with different methods of activation of M1 may also be useful in pain management. Alteration of cortical excitability occurs during the realization of motor tasks which require some type of motion component. In addition, also motor learning (ML) leads to direct activation of M1 [21], [22]. This has been demonstrated by studies involving microstimulation in animals [23], [24], imaging exam assessments in humans [25], [26] and the evaluation of cortical excitability by non-invasive brain stimulation techniques [27], [28].

Although the recent studies on invasive and non-invasive brain stimulation have provided robust data supporting the critical role of motor cortex modulation for pain control, it is not clear whether other behavioral tasks aiming at motor cortex modulation would induce similar effects. Therefore, our study intends to increase our understanding on the role of M1 modulation for the modulation of pain perception by testing the hypothesis that pain perception can be modified with different motor tasks and whether these tasks produce changes in the M1 excitability as assessed by transcranial magnetic stimulation (TMS) in healthy male individuals.

## Methods

### Study Design

We conducted a blinded (assessor was blinded to subject's intervention, and subjects were not aware to expected outcomes), randomized (randomization in blocks of 3 with a random list), controlled (right hands as within-individual control), cross-over (every subject completed three interventions in a random order) trial to determine effects of different motor tasks on pressure pain threshold and M1 excitability in healthy male volunteers. This study conformed to the ethical standards of the Declaration of Helsinki and was approved by the local IRB (Spaulding Rehabilitation Hospital, Boston, MA, USA). All participants read and signed written informed consent before initiating any study procedures.

### Subjects

Fifteen healthy right-handed male subjects (mean age: 25 years, SD 7.69, range: 19–42 years) were recruited from general population by postings in universities, internet and public places in the Boston area. Interested individuals were screened for eligibility by phone. Subjects were eligible to participate if they fulfilled the following criteria: (1) age between 18–45 years; (2) right-handed; (3) male; (4) no rheumatologic disease; (5) no clinically significant or unstable medical or neuro-psychiatric disorder including chronic pain disorders (as assessed by a checklist); (6) no history of alcohol or substance abuse within the last 6 months; (7) no contraindication to TMS; and (8) no use of central nervous system-effective medication. All study participants provided written, informed consent. All study participants provided written, informed consent. Since the menstrual cycle and age play a role in cortical excitability [29], [30], [31], we only recruited male subjects younger than 45 years of age to control for these effects. Additionally, we only included right-handed subjects in order to avoid motor ceiling effects and to ensure significant learning effects with these tasks as we trained the non-dominant left hand [32], [33].

The sample size calculation was based on a study also assessing pain threshold levels in healthy volunteers after targeting M1 excitability [34]. We assumed a type I error of 5%, a type II error of 10%, and a power of 90%, and a mean difference of 8% (±6%) in the active group and a difference of 1% (±1%) in the other groups. Using a sample size calculation for normal distribution, eight volunteers would be necessary. However given attrition rate and other unexpected factors including also the addition of a third comparison group we increased the sample size to total of fifteen subjects.

### Experiment

Subjects completed a total of three study visits. There was an interval of at least one day between each visit to avoid carryover effects. Every visit included one of the three different tasks (see below), which were performed with subjects' left hand, each for 20 minutes. The right hand served as a control as there were no tasks performed with this hand. Participants were randomly assigned to a given order of interventions. Before and after the intervention, pressure pain threshold levels were determined for both hands; thus as mentioned before, the results of the right hand served as the control group for each individual to avoid inter-individual variability. Additionally, the visits included several assessments scales and tasks (see below) as well as TMS measurements before and after the intervention.

### Motor Tasks

Participants were requested to perform the following tasks in a randomized and counterbalanced order:

#### Motor Learning with Visual Feedback (ML_sighted_)

This task consisted of four 5 min blocks of tracing a set of shapes and words, which were shown to subjects on a computer screen. Tracing was performed on an electronic board using an electronic pen with the left hand. To enhance learning, the complexity of shapes and words were increased over time. ML component was measured by comparing the number of completed shapes and words between the first and last block.

#### Motor Learning without Visual Feedback (ML_blindfold_)

Subjects used the left hand to draw different shapes (triangle, square, and pentagon). As subjects were blindfolded, the shapes were marked with pins at each corner to give feedback of where the angles of the shape were located. To enhance learning, the difficulty of shapes was increased over time. ML was measured by counting the total amount of completed shapes comparing the first and last sequence.

#### Motor Activation with Simple movements (MA_simple_)

Participants were requested to perform sequences of different simple hand movements (opening/closing, pronation/supination, flexion/extension of the wrist, abduction/ adduction of fingers). M1 activation and attention were ensured by alteration and increased complexity of movements over time [35], [36].

### Pain Assessment

Pressure pain thresholds were the primary outcome of this study and assessed with an electronic algometer (J Tech Medical Industries, USA) before and after the intervention for both hands. The device had a 1-cm^2^ hard-rubber probe, which was pressed against the thenar surface of the hand. The investigator, who assessed the pain threshold levels (MSV), was trained to this procedure and blinded to the intervention and not able to view the display of pressure intensities. Subjects sat comfortably in a chair, with arms placed on the arm rest, and instructed to say when the stimulus became painful and to be consistent with notification of painful sensation. This procedure was repeated 3 times.

### Transcranial Magnetic Stimulation (TMS)

Only one trained assessor (MSV), who was blinded to the intervention, conducted TMS measurements in all subjects to ensure a homogeneous assessment. TMS measurements (secondary outcome) were performed with a Bistim^2^ stimulator (Magstim Company LTDA, UK) and a commercially available 70 mm figure-of-eight coil. Responses to stimuli applied to the right M1 were recorded from the contralateral first dorsal interosseus muscle (FDI). Silver/silver chloride electrodes were placed over the muscle belly (active electrode) and distal phalanx of the index finger (reference electrode) to record motor-evoked potentials (MEP). A ground electrode was placed over the forearm. MEPs were amplified and filtered using a Powerlab 4/30 (ADinstruments, USA) with a band pass of 20–2000 kHz. Signals were fed to a personal computer for off-line analysis using data collection software Scope and LabChart (ADinstruments, USA). All measurements were performed immediately before and after the intervention.

TMS measurements included the determination of the resting motor threshold (RMT), which was defined as the lowest intensity eliciting a MEP of at least 100 µV in 3 out of 5 trials. Fifteen MEPs at intensity to elicit MEPs of at least 0.5 mV were recorded. Paired-pulse technique was used to record short intracortical inhibition (SICI) with interstimulus interval (ISI) of 3 ms, and intracortical facilitation (ICF) with ISI of 10 ms. First subthreshold- conditioning stimulus was set at 70% of RMT and second suprathreshold- test stimulus at the intensity to elicit a MEP of at least 0.5 mV. Recordings were made in random order having a total of 45 recordings (including test stimuli). Between each pulse we set an interval of approximately 7–10 seconds. Off-line analyses included measures of peak-to-peak amplitude and absolute integral. Additionally, 30 cortical silent periods (CSP) at 110%, 120% and 130% of RMT were elicited in a random order. CSP comprised a single TMS stimuli delivered during isometric voluntary contraction (10% of maximal contraction) of the left APB muscle. Off-line analyses measured the duration of each relative CSP.

### Other Assessments

Other assessments and outcomes comprised the following: Purdue Pegboard test (PP) [37], Visual Analogue Scale (VAS) for anxiety, and Go-no-go test (GNG) adapted from the study of Nosek and Banaji (2001) [38]. The idea of testing go-no-go task is that chronic pain is associated with significant emotional and cognitive changes and although we studied healthy subjects only, this task can identify changes in mood, and in affective and cognitive abilities [39], [40]. Subjects performed three blocks of 30 words with 10 correct answers of either one meaning. In each block mixed words were presented with positive, negative and neutral meaning (e.g. well, death, mineral). Words were displayed at a computer screen with white background. Interstimulus time between each word was set at 1500 ms. Within each block, subjects were instructed to respond by pressing a button (GO stimulus) only if the displayed words had a valence meaning, what was defined for each sequence (1.block: negative, 2.block: neutral, 3.block: positive). This test measures the ability of affective processing.

### Statistical Analyses

Analyses were done with GraphPad Prism version 4.00 for Windows, (GraphPad Software, USA). To assess the learning component of the two tasks ML_sighted_ and ML_blindfold_, paired two-tailed t-tests were performed comparing first and last sequences of each task.

Changes in pain threshold levels were analyzed with ANOVA for factors hand (left/right) and task (ML_sighted_, ML_blindfold_, MA_simple_). Post-hoc comparisons were done comparing before and after the different tasks using paired two-tailed t-tests. Additionally, t-tests were performed for the control groups (pain threshold of right hand) for every motor task.

For TMS data, we performed a mixed ANOVA model in which the dependent variable is the measurement of cortical excitability (RMTs, MEPs, SICIs, ICFs, CSPs) and the independent variables are the tasks (ML_sighted_, ML_blindfold_, MA_simple_) and time (pre, post) for each experiment. Moreover, one-way ANOVAs were performed for change in MEP amplitude/integral over time versus task to reveal differences among the task. When appropriate, post hoc comparisons were carried out using Bonferroni's correction.

The results of the exploratory GNG before and after the intervention were described in means and SD for amount of errors, omissions and correct answers. Furthermore, we compared differences in errors, omissions and correct answers using two-tailed, paired t-tests for pre-/post-comparison.

Following descriptions of statistical significance refer to a p-value<0.05, and a statistical trend is considered to indicate a p-value<0.1.

## Results

No adverse effects were experienced throughout the entire procedure including assessment with single and paired-pulse TMS. Of the 15 subjects enrolled, 14 completed all three visits, thus, one subject dropped out, because of scheduling difficulties. We did not include his data, as he only completed one of three visits as this was a cross-over trial.

### Learning effect for the motor learning tasks

**Figure 1:**
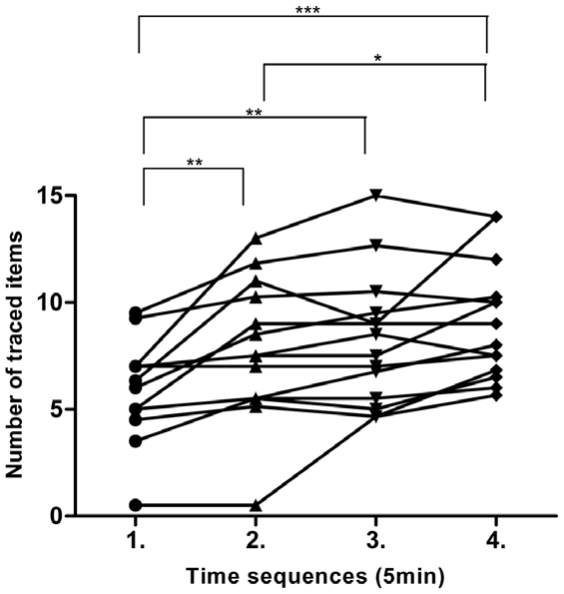
Behavioral results of task ML_sighted_. Lines indicate individual subjects' performance (number of traced words/shapes) over time. * = p<0.05. ** = p<0.01. *** = p<0.001.

**Figure 2:**
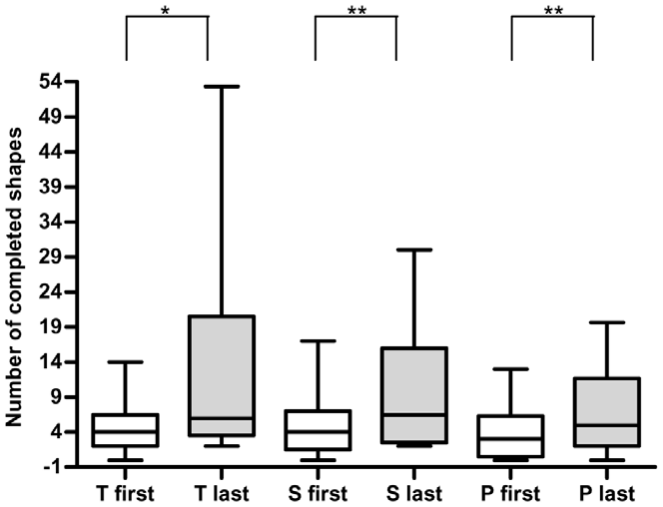
Behavioral results of task ML_blindfold_. Box-and-whisker plots show amount of completed shapes of the first and last sequence of each shape. T = Triangle; S = square; P = Pentagon. * = p<0.05. ** = p<0.01.

Sequences comparison showed significant results for both ML tasks: comparing 1^st^ to ^4th^ block of 5 min (p = 0.0003), 1^st^ to 3^rd^ 5 min (p = 0.0014), 1^st^ to 2^nd^ 5 min (p = 0.0036) and 2^nd^ to 4^th^ 5 min (p = 0.0109) of ML_sighted_ (Figure 1), and comparing first and last sequences of ML_blindfold_ (triangle: p = 0.0250; square: p = 0.0052; pentagon: p = 0.0024) (Figure 2). All participants improved their performance on the tasks sequence over time, which indicates that successful learning had occurred.

### Pain threshold across the three motor tasks and both hands (left trained and right untrained control hand)

**Figure 3:**
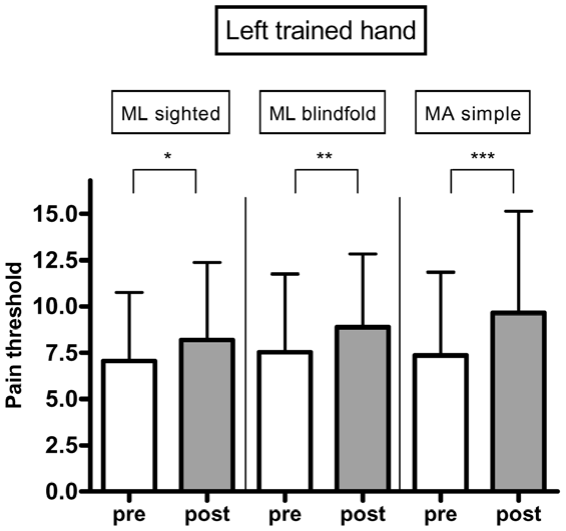
Results of pain thresholds: left hand. Pressure pain threshold levels before and after the tasks for the left trained hand. * = p<0.05. ** = p<0.01. *** = p<0.001.

**Figure 4:**
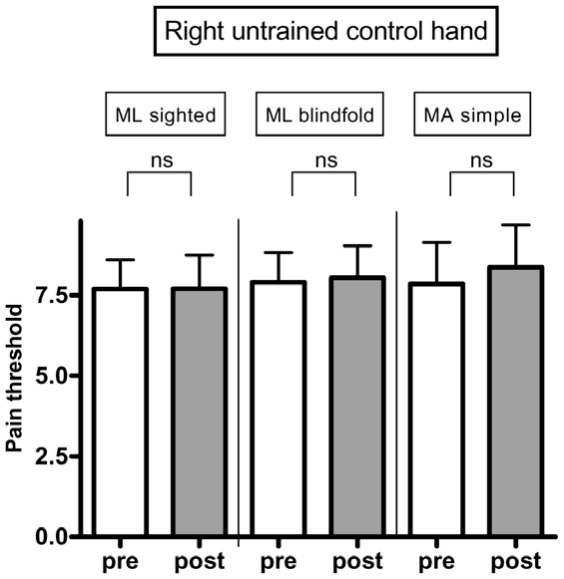
Results of pain thresholds: right hand. Pressure pain threshold levels before and after the tasks for the right untrained control hand. Ns = not significant.

We initially conducted an ANOVA with two factors (motor task and hand). This analysis showed only an effect for hand (differential effect when comparing left trained vs. right control hand, p = 0.0001). We therefore conducted separated models for each hand. For the left trained hand, there was a similar increase in pain threshold across the three motor tasks. Analyses showed a significant increase in pain threshold of ML_blindfold_ (p = 0.0037, threshold increase of 17.96%), ML_sighted_ (p = 0.0182, threshold increase of 15.85%), and MA_simple_ (p = 0.006, threshold increase of 31.24%). Although the simple task had a larger magnitude increase in pain threshold, this was not statistically significant (p = 0.15) when compared to the other two motor tasks (Figure 3). We then conducted the same analysis for the control hand (right untrained hand). The results showed that there was no significant change in the control hand for these three tasks (ML_blindfold_: p = 0.5773, threshold increase of 1.81%; ML_sighted_: p = 0.9821, threshold increase of 0.15%, MA_simple_: p = 0.1356, threshold increase of 6.53%) (Figure 4). This indicates that the effects of pain alleviation were side specific and thus induced by the motor tasks.

### TMS

ANOVAs revealed a significant difference in MEP amplitude change across motor tasks (p = 0.0233), and a trend for change in MEP integral (p = 0.0697). This indicates that there was a difference in M1 excitability changes across the tasks. P-values and means/SD for post-hoc comparisons for all TMS measures are shown in Table 1. Comparing MEP, ICF and CSP before and after the intervention, significant effects could be revealed mainly for MA_simple_ showing that cortical excitability increases after this task (increase in MEP and CSP shortening). SICI and RMT did not change in all tasks.

**Table 1:** Results of transcranial magnetic stimulation (TMS) measures.

Transcranial Magnetic Stimulation Measurement	Motor Tasks		
	ML_sighted_	ML_blind_	MA_simple_
**MEP Amplitude**	1.37 [±0.32] 1.27 [±0.35]	1.39 [±0.25]1.4 [±0.28]	* 1.28 [±0.23] 1.49 [±0.35]
**MEP Integral**	11.68 [±3.88] 11.13 [±4.08]	12.2 [±2.92] 12.67 [±4.12]	* 11.57 [±3.28] 14.13 [±4.95]
**ICF Amplitude**	1.027 [±0.188] 1.037 [±0.205]	1.013 [±0.179] 1.064 [±0.169]	**∧** 0.871 [±0.18]0.982 [±0.223]
**ICF Integral**	0.977 [±0.178] 1.012 [±0.255]	0.986 [±0.176] 1.011 [±0.215]	* 0.795 [±0.191] 0.977 [±0.269]
**SICI Amplitude**	0.244 [±0.173] 0.268 [±0.236]	0.216 [±0.160] 0.211 [±0.146]	0.316 [±0.168] 0.292 [±0.222]
**SICI Integral**	0.249 [±0.248] 0.186 [±0.212]	0.211 [±0.141] 0.213 [±0.137]	0.279 [±0.169] 0.259 [±0.197]
**CSP 110%**	* 90.82 [±21.9] 79.43 [±28.97]	86.15 [±31.59] 83.33 [±27.36]	* 89.68 [±27.28] 79.18 [±24.54]
**CSP 120%**	114.99 [±20.1] 108.36 [±26.39]	117 [±33.18] 109.83 [±34.7]	**∧** 116.66 [±27] 107.71 [±29.98]
**CSP 130%**	137.14 [±19.35] 129.27 [±29.44]	137.99 [±32.7] 130.96 [±38.43]	* 137.29 [±29.3] 125.11 [±30.81]

Values before and after the interventions are given as mean [± standard deviation]. MEP amplitude in mV; MEP integral in mV*ms; SICI and ICF in their index; CSP in ms. Student's t-test for pre/post-comparison. Statistical significance (p<0.05) indicated with asterisk *. Statistical trend (p<0.1) indicated with ∧ .

MEP: motor evoked potential; ICF: intracortical facilitation; SICI: short intracortical inhibition; CSP: cortical silent period; ML: motor learning; MA: motor activation; SD: standard deviation.

### Further Assessments

The mean of PP - control task for motor function changes - for the left hand before intervention was 13.51 (SD 1.46), and 13.63 (SD 1.46) afterwards. T-test for pre-/post-comparison did not show significance (p = 0.4112). Similar results were obtained for right hand (mean-before: 14.33, SD 1.51; mean-after 14.62, SD 1.26; p = 0.09).

No changes in mood, affective and/or cognitive capability were present as indicated by GNG and VAS. The t-test for VAS for anxiety showed no differences (p = 0.84) between pre-/post-comparison. For the GNG, there were no differences in the amount of correct answers (p = 0.95; pre = mean: 27.37; SD 1.98; post = mean: 27.39; SD 1.97), omissions (p = 0.95; pre = mean: 2.61; SD 1.99; post = mean: 2.59; SD 2.09) and errors (p = 0.36; pre = mean: 3.93; SD 1.81; post = mean: 4.24; SD 2.45) between pre-/post-comparison.

## Discussion

In this study, we showed that three different motor tasks increase pressure pain thresholds in healthy volunteers and accordingly might alleviate perception of pain sensations. This effect was specific for the hand being tested as the control hand did not show any significant difference. Interestingly, although the three motor tasks induced similar behavioral pain effects, they induced different changes in motor cortex excitability. This result suggests that motor training induced pain threshold changes may be mediated through different neural mechanisms as it is motor task specific.

Our results confirm the notion that motor learning can modulate pain threshold. Both motor learning tasks confirmed that a significant learning has taken place (behavioral improvement) and we measured a significant increase in pain threshold of 15.85% for ML_sighted_ and 17.96% for ML_blindfold_, whereas the control condition (untrained hand) showed very small pain threshold increase of 0.15% and 1.81%, respectively. Our findings are in line with other studies, which encourage cognitive effort during motor training in pain rehabilitation [28], [41]. Even though, our observations were gained in healthy subjects and may be different in chronic pain patients. In fact EEG- and PET- imaging studies support the beneficial effects of cognitive involvement during execution of complex movements [35], [42]. One interesting finding was the result of ML_blindfold_ condition - as it was also significant and even larger compared to ML_sighted_ (though this difference was not significant). In fact, one possibility that needs to be explored further is whether visual deprivation can enhance the effects of motor learning on pain modulation as visual deprivation can divert attention toward other senses [43], [44].

Despite the effects of motor learning on pain threshold, we also found that simple hand movements without a learning component were sufficient to increase pressure pain thresholds. This is in line with previous findings, which showed that motor practice is effective to alleviate pain [11], [12], [13], even if there is no combined learning or highly cognitive demand [45].

One possible limitation we could not control is that the obtained results could have been caused by peripheral effects. Such possible peripheral effect may be altered muscle activity after the exercise of simple hand movements or changes in blood flow or a different blood oxygenation level, which could have influenced the perception of pain [46], [47]. However, since all motor tasks, of which each task activated different muscles and also to a different extent, changed perception of pressure pain and varied in changes of cortico-spinal excitability as measured by TMS, it is less likely that analgesic effects result from peripheral mechanisms.

Also possible limitations due to the cross-over design and potential carryover effects need to be mentioned, as it would be possible that the motor tasks changed M1 plasticity for extended period of time [48]. To avoid these problems, we randomized the sequence of tasks which subjects completed and also designed each motor task differently to avoid carryover effects due to motor learning.

Evidence from previous studies showed that hand movements lead to an increase in cortical excitability indicated by an increase in MEP's amplitude and integral [45], [49], [50], [51]. Furthermore, Roosink et al. (2010) suggested that a complex task leads to a larger corticospinal excitability increase in comparison with simple task [35], [36]. Though in this study, the complex task cannot be compared to our motor learning task.

One result that needs to be further discussed is that MA_simple_ led to larger effects on cortical excitability compared to ML tasks. There are several reasons to explain lack of cortical excitability changes in the ML condition. One reason would be associated with the time course of excitability changes. A recently published study showed that MEP does not further increase even after a retention period of motor training, thus, suggesting that the M1 changes in a dynamic time course due to a motor-driven demand [52]. This could explain why the three motor tasks in our experiments changed cortical excitability differently. Nevertheless, the comparison of changes in cortical excitability between studies investigating the effects of ML or simple movements is limited as studies used different paradigms. Firstly, the duration of task execution has a significant effect on M1 activation as shown by a study of Muellbacher et al. (2001) [53]. In this study significant differences between 30 and 60 minutes could be seen; in contrast, in our study subjects conducted each task for 20 minutes. Secondly, examining studies that reported increase in MEP due to ML, it shows that tasks involved fast thumb repetitive abduction movements [54], whereas the task in our study might have involved a larger cognitive demand. Indeed “learning” component is differently defined and goes from “learn” how to move a finger faster to “learn” how to move the hand to reach a certain goal as it was mandatory in our experiments [49], [53]. One important aspect here is that our motor learning task might have engaged other neural circuits and in fact the effects of our ML task on pain threshold might be due to the engagement of these other neural networks. For instance, ML in this study might have activated also working memory-related cortical areas. We showed in a previous study that modulation of the dorsolateral prefrontal cortex (DLPFC) leads to an increase in pain threshold in healthy subjects [34]. Thus motor learning tasks might change pain perception through activation of non-motor neural networks as evidenced in this study.

As we show in this study that simple hand movements without a complex cognitive component is effective to modulate pain and change M1 excitability, this finding might be interesting for the field of physical medicine and rehabilitation and the novel rehabilitation techniques with robotics, as some of these techniques use more repetitive movements; however in some cases in a more passive way [55], [56]. As passive movements of upper extremities seems to be sufficient to activate M1 and therefore induces changes in cortical plasticity [54], this intervention might also have a significant impact in pain modulation. In fact these results might extend to mental imagery and motor observation. For instance, imagination of amputated arm movements in chronic phantom pain can induce analgesic effects, which further supports our hypothesis and highlights the clinical relevance of the present findings [57], [58]. Seidel et al. (2011) showed an average pain reduction of 2.8 in VAS after mirror visual feedback therapy in phantom limb pain. Similarly Mercier et al. (2009) showed a reduction in 38% in pain as indexed by VAS in phantom limb pain patients. [57], [58]. Nevertheless, it is important to underscore that the lack of changes in cortical excitability in motor learning task does not imply that simple movements are better than tasks with a learning component to modulate pain. Further studies are needed to investigate and compare the effects of different motor tasks in patients with chronic pain.

None of the tasks reduced SICI significantly, thus, we could not confirm the reduction of SICI as described by Liepert et al. (1998) [50], even though we found a tendency to reduce SICI [51]. A reason for that might be the different design of the tasks as already discussed. Since the task in their study used fine motor control of small hand muscles compared to our task involving multiple hand muscles, a broader cortical representation area might have been activated which therefore resulted in different inhibition patterns. Interestingly, the study of Rosenkranz and Rothwell (2006) could also not reproduce a significant reduction of SICI, which is in line with our findings [54]. In contrast, we revealed significant increase in ICF for one of the tasks (MA_simple_), which is in line with the concept proposed by Ziemann et al. (1996) that inhibitory and excitatory neuronal circuits likely act independently [59].

Finally, the most prominent changes besides MEP changes occurred in CSP. Overall, there was a common tendency that CSPs with different intensities of 110–130% decreased in all tasks, and it could reach significance in ML_sighted_ (but only for 110% for this task) and MA_simple_. Previous evidence suggested that CSP and SICI have different underlying mechanisms, and that those of CSP can be linked to GABA-B receptor-mediated inhibitory neurotransmission [60]. Furthermore, CSP is evoked through direct stimulation of interneurons in M1 [61], and it is likely that CSP is caused by a decrease in cortical firing induced by activation of inhibitory interneurons. Based on that evidence which suggests that CSP has a cortical origin [62], [63], [64], it is most likely that excitability of cortical neurons activated by simple hand movements is increased mainly by disinhibition [65]. In fact Lefaucher et al. have shown that patients with chronic pain have a defective inhibitory activity as indexed by CSP and ICI as compared to healthy subjects [3].
